# Health assessments of refugee minors arriving in Norway – a modified Delphi study among health professionals in primary care settings

**DOI:** 10.1186/s12889-024-19871-2

**Published:** 2024-09-12

**Authors:** Cecilie Dangmann, Annette Løvheim Kleppang, Marja Leonhardt

**Affiliations:** 1 Faculty of Social and Health Sciences, Inland Norway University of Applied Sciences, Hamarvegen 112, Elverum, NO 2418 Norway; 2 Norwegian National Advisory Unit on Concurrent Substance Abuse and Mental Health Disorders, Innlandet Hospital Trust, Brumunddal, 2381 Norway; 3 Faculty of Health Science, VID Specialized University, Oslo, Norway

**Keywords:** Refugee, Minor, Health assessment, Resettlement

## Abstract

**Background:**

Refugee minors are considered particularly vulnerable to negative health consequences from war, flight and resettlement. Offering health assessments after arrival in a host country could uncover unmet health needs and provide access to treatment. In Norway, a national guide describes these assessments, but little is known about its implementation especially for refugee minors. Thus, the aim of this study was first to explore how health assessments of refugee minors are carried out, second how health professionals perceive the needs of refugee minors and third, the competencies they perceive as necessary to meet the needs of refugee minors.

**Method:**

A modified Delphi study in three rounds was conducted using online surveys and one focus group to collect data on the needs and resources of refugee minors, essential factors for a good and health assessment practice. Participants were 54 health professionals responsible for early health assessments of refugee minors, throughout the Norwegian municipalities, working in primary care settings. Quantitative data was analysed descriptively, and qualitative data with content analysis.

**Results:**

Health assessments of refugee minors were predominantly conducted by public health nurses, but the organisational structures surrounding assessments varied greatly according to the size of the municipalities and to how much resources were allocated. The feeling of safety was found to be paramount to ensure a good start in a new country for refugee minors. The top four competences professionals should have, were ‘general communication skills’, a ‘health professional background’, ‘expertise in children’s health’ and ‘knowledge about the national guide’. To ensure good health services for refugee minors, improved, more comprehensive, and mandatory directives for children and young individuals was highlighted.

**Conclusion:**

Although most refugee minors were invited and attend health assessments, one third of participating municipalities did not offer health assessments to all newcomers and the organisation and content of the assessments were diverse. Several topics, especially mental health, were postponed or not routinely addressed, contrasting with current knowledge of unmet health needs for this group. Missing documentation, practical barriers and providing general health information took time away from doing the actual assessments. The perceived needs of refugee minors were safety and stability, combined with meaningful activities, thus a coordinated effort from several services is necessary. Suggestions for improvements were more time given to assessments, better organisation and co-operation, improved competence and guidelines adjusted for age.

## Background

At the end of 2023, the worldwide number of forcibly displaced people reached an unprecedented 117 million. The majority of them are internally displaced or hosted by neighbouring countries, most of which are low- and middle-income countries close to zones of armed conflict [1]. Half of the worldwide displaced population and about one third of asylum seekers in Europe, are children under 18 years [1, 2]. After the Russian invasion of the Ukraine in February 2022, more than 6.5 million Ukrainians fled to other countries in Europe, many receiving collective temporary protection [3]. More than 80 000 Ukrainians have arrived in Norway, a third of them being children [4]. The term refugee minor refers to a person who is forced to move and unable to return safely and is under the age of 18 years, as proposed by the United Nations [5]. In this study we use the term broadly to include asylum seekers and family members granted residence, as well as those granted status through UN applications, as they all have the same rights to health care and health assessments in Norway.

Forced migration is seen as a global threat to health, and refugee minors are considered to be particularly vulnerable. The context of displacement affects all parts of their life happening at a crucial time of their physical, emotional, social and cognitive development [6]. Refugee minors are uprooted from their home, friends and family, some undertaking long and hazardous journeys or spending prolonged periods in transit or camps. Many have experienced potentially traumatic events such as war, disasters, violence and death, as well as a lack of basic resources such as shelter, food and healthcare [7]. Although resettlement in another host country means safety and new opportunities, it also involves new challenges as they have to adjust to a new life somewhere else [8]. High levels of mental distress among refugee minors are well documented, although this is not consistent across all groups [9]. Unaccompanied refugee minors are considered particularly vulnerable [8, 9]. Consequences on refugee minors’ health are therefore not only immediate, but also affect their development, future health and wellbeing [6].

Studies on health assessments after resettlement describe how these assessments uncover a variety of health problems needing treatment or follow-up: Infections, sleep or behavioural problems, growth impairment, undiscovered hearing and visual impairment, untreated caries, or vitamin D deficiency [10–12]. Lack of health services prior to flight means some have not received recommended childhood vaccinations and are more vulnerable to infections [9]. A recent scoping review found that migrants’ healthcare needs are largely unmet due to multiple barriers in accessing healthcare services. Examples of such barriers were legal restrictions, financial hardship, discrimination, language problems or lack of knowledge of healthcare systems [13]. Offering early health assessments could be one way to improve early access to appropriate health care.

Systematic health examinations directed at newly arrived refugee minors are implemented in most European countries. Screening for communicable disease, most commonly tuberculosis, is often mandatory, whereas broader assessments of minors’ individual health needs are often voluntary [14]. The content of a voluntary health assessment varies from country to country, but typically consists of a review of the child’s health history, a physical examination, and to some extent screening for mental health problems. Identification of communicable diseases and immunisation status will also be included. Referral for treatment and complimentary healthcare is provided as necessary [14]. Health assessments traditionally focus on physical health, whilst evidence indicates that mental health should also be prioritized [8]. A recent study from Denmark showed that 88% of refugees attended such voluntary health assessments, and 64% of these had one or more health needs that required further testing, treatment or follow-up [11]. Undetected and untreated health needs can have long-term consequences for their health, wellbeing, education and integration. Early, comprehensive, and tailored assessments including mental health assessments done by a paediatric nurse or a physician, are therefore necessary and recommended [10, 14, 15].

In Norway, all residents and asylum seekers have the same rights to healthcare and at no cost for children [16]. A national guide describes the right to equitable health services for newly arrived refugees, asylum seekers and reunited families [16] and it recommends that the first broad health assessment should take place soon after arrival to the host country, followed by a second assessment about three months later. For the first assessment, the guide lists 12 conditions to assess, including acute and chronic conditions, infectious diseases such as tuberculosis and symptoms of Post-Traumatic Stress Disorder (PTSD). The guide provides standardized forms (short and long versions, and versions for accompanied and unaccompanied minors under the age of 18 years) to be used during the assessment. The forms have questions and prompts to assess physical and dental health, and 10 standardized questions on traumatic experiences and psychological symptoms. All versions of the forms specify that these 10 questions are voluntary to answer and should only be asked if deemed relevant, but it is not stated if these questions are based on any validated screening instruments. In the version for minors, it is recommended to rephrase the questions on psychological health, leave out some of the questions, or only ask these questions to parents to protect the child. For the three-month assessment, the guide suggests various tests, assessment of vaccination status and recommends a list of health topics to address. It provides another form with questions on general health which includes a checklist with nine questions on traumatic experiences (based on the Harvard Trauma Questionnaire [17] and PTSS-10 [18] and eleven questions on psychological symptoms with a suggested cut-off for referral. Again, the form specifies that the psychological screening should only be used on indication. Notably, this form has no adjusted versions for children. The guide suggests that health assessments can be done by either a physician, a nurse or a public health nurse (PHN^1^), and that it should be recorded by the health services and that a paper copy of the assessment should be given to the person.

The Norwegian guide describes how health assessments should be done, but we know little about how they are actually carried out for refugee minors. Personnel doing these assessments will also have close-hand experience of what the current needs are among newly arrived refugee minors and if these assessments are relevant to their needs. The overall aim of this study is therefore to explore the health assessments of refugee minors in Norway. More specifically, we aimed to (1) explore the current practice of health assessments among refugee minors, (2) explore how frontline health professionals perceive the current needs of refugee minors, and (3) explore what competencies and tools health professionals perceive as necessary to meet the needs of refugee minors.

## Methods

### Study design

**Fig. 1:**
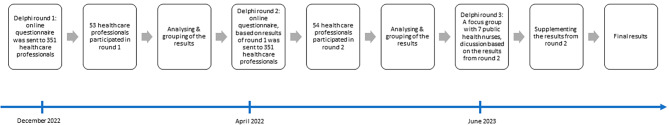
Flowchart of the Delphi process

In this study we used a descriptive design, applying a Delphi technique, which is an appropriate method to collect subjective statements on a collective basis, on topics where there are no prior existing true consensus [19]. The goal of a Delphi technique is to gain a group consensus through collecting a series of expert opinions, and several varieties of the method has been implemented across several studies and disciplines [19]. According to Hasson et al., the method is flexible, but recommend the following four main steps: (A) Survey development (including piloting among a small group of people), (B) participant recruitment, (C) Data collection and analysis (various rounds; in the second and subsequent rounds participants are asked to rank or respond to analysed options from the first round) and (D) Ending the Delphi process when an acceptable level of consensus has been reached [20]. We used a modified Delphi technique in which we conducted the first two rounds as an online survey and the third round as a focus group interview. In the latter we deviated from an ordinary Delphi technique, which stipulates anonymity of the respondents [21]. We have chosen to deviate from a classical Delphi technique as it does not allow for participant discussion or for them to elaborate their views and we therefore included focus group interviews as round three. Figure 1 illustrates the Delphi process used in the present study.

### Sample and procedure

The experts in this Delphi process were defined as health professionals employed in the primary health care sector, potentially working with or responsible for refugees’ health. This included PHNs in schools, healthcare centres or refugee healthcare centres, and municipal chief physicians and nurses. Potential participants were identified via official contact information available on their respective municipal websites. This procedure resulted in a distribution list of 351 participants, representing municipalities of all sizes and from all of the eleven Norwegian counties.

The first two rounds were conducted as an online questionnaire which was programmed with the web-based survey tool “Nettskjema”, which offers security measures to ensure data accuracy and privacy appropriate for research [22]. The questionnaires were developed by the authors based on the research questions and the major topics of health assessment described in Chap. 4 of the Norwegian National guide for asylum seekers, refugees and reunited family members [16]. Both questionnaires included demographic questions such as county, professional background and municipality size. Answers could be chosen from a drop-down menu. In the first questionnaire (round 1) participants were asked open-ended questions on the needs and resources of refugee minors, and which factors they meant were essential to provide a good start in the host country. In addition, they got open-ended questions related to health assessment practice: Who was responsible, how the health assessments were conducted, and which tools were used. The questionnaire was tested among five PHN who were either employed in a refugee health service or previously worked with refugees and were presently working at the nursing department of a Norwegian university. The questionnaire was then sent to 351 health professionals employed in the primary healthcare services throughout Norway via e-mail by the last author. After one week, a friendly reminder was sent to the same distribution list. Fourteen days after the first e-mail, the online survey was closed for answers. We received data from 53 respondents (15%) representing all Norwegian counties. Qualitative content analysis was used to analyse these answers, based on the descriptions of Graneheim and Lundman [23]. All authors contributed to the interpretation of the data. A summary of results and more specific questions on health assessments were included in a second online questionnaire (round 2) to the same 351 persons. Fifty-four health professionals from all the eleven counties participated in round 2. Respondents could provide their e-mail-address if they were interested in participating in the focus-group interview (round 3). Results from round 2 were analysed and presented by the first and last author to the focus group who elaborated on the findings. This interview was conducted digitally on Microsoft Teams with seven PHNs, working in schools, health care centres or refugee reception centres. The first author led the discussion, and the last author took notes. The interview was audio taped, transcribed verbatim and analysed with qualitative content analysis [23]. The first round (online survey 1) was sent out in December 2022 and answering the questionnaire took approximately 10 to 15 min, whereas the second round (online survey 2) from April 2023 took 15–20 min. Round 3, the focus group interview was conducted in June 2023 and lasted one hour.

### Ethics

Participation in the online surveys (round 1 and 2) was voluntary and anonymous, if respondents did not themselves provide an email (round 2). Participants signed an electronic informed consent from within “Nettskjema”. The study was approved by the Norwegian Agency for Shared Services in Education and Research (SIKT) with the reference number 778056.

## Results

Our study population in the online survey in Delphi round one and two was 53 and 54 participants respectively. Most of them were PHNs, followed by physicians. Participants represented all the eleven Norwegian counties. The sample in the third round, a focus-group, comprised seven PHNs, representing five different counties. Table 1 presents the description of the entire sample.

**Table 1:** Participants in round 1–3

	Round 1	Round 2	Round 3
Total	n = 53	n = 54	n = 7
**Profession**			
PHN/nurse	34 (64%)	45 (83%)	7 (100%)
Medical doctor	11 (21%)	5 (9%)	
Other	8 (15%)	4 (8%)	
**Geographical spread**			
Mid- and Northern Norway	13 (25%)	16 (30%)	2 (29%)
Western Norway	13 (25%)	11 (20%)	
Southern and Eastern Norway	27 (50%)	27 (50%)	5 (71%)
Size of municipality*			
Small	4 (8%)	9 (17%)	2 (28%)
Medium	19 (36%)	20 (37%)	2 (28%)
Large	30 (56%)	25 (46%)	3 (44%)

Note: * Size of municipality: Small (less than 5000 inhabitants),* medium (5000–20 000 inhabitants)*,* large (more than 20 000 inhabitants)*

### Health assessment of refugee minors – current practice

#### Responsibilities

From the first Delphi round we identified three models of organizing responsibility for the health assessment: (1) a refugee health team consisting of a nurse (often PHNs), a physician and other health professionals such as physiotherapists or psychologists, often within a municipal health service designated for refugee or migration health; a model more common in larger municipalities, (2) a team of one nurse (often PHNs) and a physician with shared responsibility, either doing the assessments together or in separate consultations, and (3) one person responsible for all assessments. PHNs were often responsible, several had part of their position designated for health assessment of refugee minors, but some municipalities had delegated the task to General Practitioners’ (GP) offices. A small number of respondents said that health assessment of refugee minors could not be offered due to the lack of routines or personnel. When it comes to cooperating with other services, for example for follow-up or referral, other health personnel in dental, psychiatric or specialist health services were often mentioned. Cooperating with municipal refugee services and personnel in reception centers (often run by private companies) was also common. Centers for unaccompanied refugee minors often cooperated with child welfare services and the police. Lastly, schools and kindergartens were stated as important collaborators.

#### Users

About 71% of the respondents stated that all refugee minors who arrived in their municipality were invited to a health assessment. The remaining third of respondents only offered assessments to families with extra needs or those who asked for help themselves. Some municipalities had no routines for informing health services of new arrivals, thus health personnel coincidentally found out who had arrived and could invite them for health assessments. 80% of our participants confirmed that almost all of those invited actually attended the assessments, very few (4%) had attendance rates below 70%. The majority (85%) always used certified interpreters in all assessments.

10% of the respondents only invited parents for health assessments, not children. However, the majority (67%) invited the whole family, and 40% did the assessment with the whole family present, whilst 20% invited the whole family but did separate health assessments while the rest of the family waited outside. If they felt the children were old enough, 20% of the respondents preferred to talk with the children alone. Participants in round 3 disagreed with the practice: *“I don’t think that it is useful to invite the parents alone. We cannot assess children’s health through the lens of a parent”*, said one participant, and another agreed: *“Children should be offered an own initial assessment*,* at least if they were over 12 years of age.”* Some argued that assessing children whilst other family members or parents were present could prevent children and youth from talking freely and that intimate topics such as sexual health may not be suitable to discuss. Some also described a different routine where they did the initial assessment with the whole family present but delegated the individual assessment of the child/adolescent to school nurses, as they will be the ones providing continuing care.

#### Health assessments content

Based on the results from round 1, the content of the health assessment was itemized and grouped into physical and mental health topics for round 2. Among physical health topics, almost all participants routinely assessed vaccination status, risk of tuberculosis, chronic illness and pain - see Table 2. A majority always measured weight and height (65%), assessed oral health, motor development or did blood tests for infectious diseases (52–62%). A third did these assessments only when a situation or symptoms indicated that there was a need for it. Examining heart and lung function or addressing sexual health topics were by most done only when indicated (73%). Respondents commented that they adjusted the content of the assessment according to the age and country of origin of the child.

**Table 2:** Physical health topics (ranked by most often addressed)

Physical health topics (*n* = 52)	Always addressed	When indicated	All referred	Referred when indicated	Never addressed
Chronic illness/ medication	51 (98%)	1 (2%)			
Vaccination status	50 (96%)	1 (2%)	1 (2%)		
Pain or discomfort	47 (90%)	5 (10%)			
Tuberculosis exposure/risk	44 (85%)	4 (8%)		2 (4%)	2 (4%)
Nutrition	43 (83%)	9 (17%)			
Self-assessed physical health	38 (73%)	9 (17%)			5 (10%)
Weight	34 (65%)	14 (27%)			4 (8%)
Height	34 (65%)	13 (25%)			5 (10%)
Oral status	32 (62%)	10 (19%)	7 (13%)	3 (6%)	
Blood test (infectious diseases)	29 (56%)	13 (25%)	1 (2%)	4 (8%)	5 (10%)
Motor development	27 (52%)	20 (38%)		5 (10%)	
Vision test	24 (46%)	18 (35%)	1 (2%)	7 (13%)	2 (4%)
Hearing test	23 (44%)	18 (35%)	1 (2%)	7 (13%)	3 (6%)
Genital mutilation	12 (23%)	37 (71%)	1 (2%)		2 (4%)
Listening to lungs/heart	12 (23%)	18 (35%)		8 (15%)	14 (27%)
Sexual health/contraception	10 (19%)	38 (73%)			4 (8%)

*Note: Two participants did not answer this question as they did not routinely do health assessments themselves, n = 52*

Among mental health topics, almost all participants routinely addressed familial and social networks, sleep and quality of life (88–98%) - see Table 3. About 77% of the respondents always assessed traumatic experiences, while topics such as behavioral problems or substance abuse were mostly discussed on indication. In round 3, participants discussed the pros and cons of assessing some topics only “when indicated”. Some found that recent arrivals from Ukraine had experienced more potentially traumatic events than those arriving only a year earlier, and therefore argued that assuming relevance of trauma purely from country of origin was impossible. Others said that since some children had missed out on many health assessments due to flight, a thorough assessment including for example vision and hearing was important, even if they had no particular problems.

**Table 3:** Psychological health topics (ranked by most often addressed)

Mental health topics	Always addressed	When indicated	All referred	Referred when indicated	Never addressed
Family network	51 (98%)	1 (2%)			
Sleep problems	47 (90%)	5 (10%)			
Self-assessed quality of life	46 (88%)	5 (10%)			1 (2%)
Social networks/friends	45 (87%)	7 (13%)			
Traumatic experiences	40 (77%)	12 (23%)			
Self-assessed psychological health	38 (73%)	12 (23%)			2 (4%)
Psychological symptoms/distress	35 (67%)	17 (33%)			
Behavioral problems	21 (40%)	30 (58%)			1 (2%)
Alcohol/drug use	14 (27%)	32 (61%)		1 (2%)	5 (10%)

(*N* = 52)

When asked if they used structured assessment tools for mental health, 48% said they used the forms and questions provided in the national guide, 27% used only parts of these forms, 14% had developed their own assessment tool and only 3% did not use any structured assessment tools. Some explained that since the guide and included forms were not adjusted for children, especially 0 to 6 years, they had developed their own or adjusted the existing ones along the way. One participant had been advised not to pose children questions on trauma. In round 3 the participants discussed the pros and cons of structured assessment tools. Some emphasized that you need knowledge and experience to use the tools correctly. They had also experienced how structured assessment tools suited some cultural backgrounds better than others, for example that structured assessments worked well with Ukrainians as a more objective way to approach psychological issues, and that with Syrians or Eritrean refugees, a less structured and more conversational assessment was considered a better approach.

In round 3 it became apparent that several municipalities differentiated assessments depending on asylum status – those awaiting settlement and living in reception centers received a shorter assessment to uncover more acute health problems, and to stabilize or prevent a worsening of preexisting conditions. Mental health was often not included in this shorter assessment, and part of the reasoning was that assessing mental distress in children required a long-term relationship and follow-up. One participant said that *“you never know how long they would stay”*, therefore mental health assessments were postponed until they had permanent residence. This reasoning was contested, others argued that time in reception centers was not short but could be between 6 months to one year, and that distinguishing between what was acute and long-term mental distress and what *“had to be done and not done”* was often difficult.

Participants described “guidance” as an important part of the health assessments and 80% always informed families about health services, answered general questions and helped with practical problems. If relevant, they also gave guidance on culture, parenting, or nutrition.

#### Documentation and referral

All health assessments were documented in electronic records, 71% of which were health records kept by preventive CHS run by PHNs. When referral was indicated – 80% transferred relevant parts of the health assessments to other services. Although being recommended in the guide, only 8% offered parents a printed copy of the assessment. For 15% of the respondents the documented assessment was unavailable to any other service. In round 1, several commented that after the initial assessment, the children were automatically transferred to CHS to follow the national preventive program. Results of round 2 showed that 40% of the initial assessments were done by the CHS themselves and no transfer was necessary. About 30% informed the CHS of the results and then transferred the children, while 25% automatically transferred the record but did not inform the CHS unless the child required additional follow-up. In round 3, participants agreed that documentation in general was complicated and very time-consuming. They often experienced difficulties in retrieving test results (e.g. tuberculosis screening) or accessing records, and had to repeat testing, transfer or trace missing records *“even though they had really tried to create a system”.*

#### Refugee minors’ needs

**Fig. 2:**
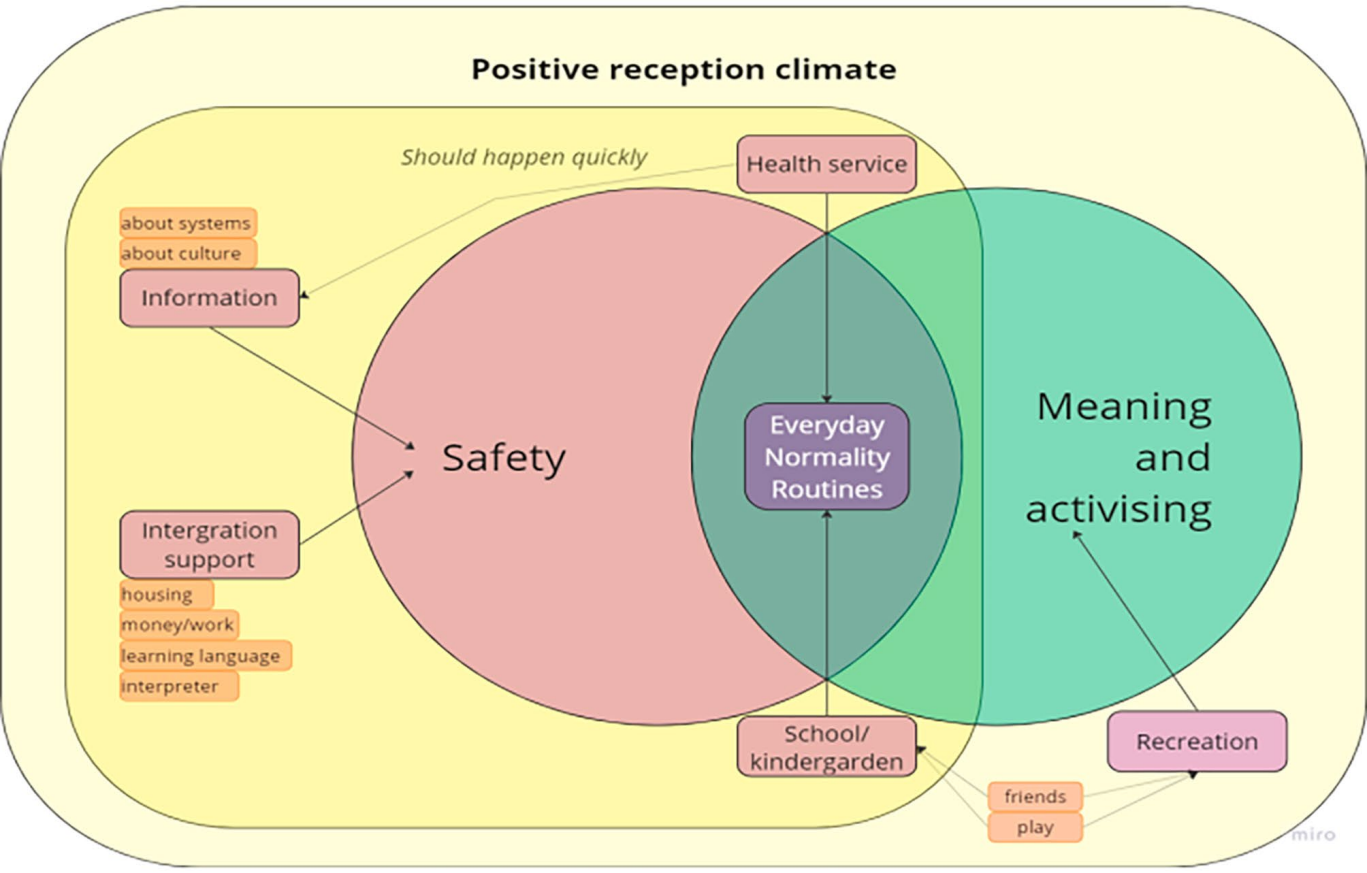
Perceived needs of refugee minors

The second aim of this study was to gather the expert opinions of frontline professionals on the needs of refugee minors. This was done through open questions in round 1. A summary of these opinions was rated for accuracy in round 2. Respondents stated that a feeling of safety was paramount to ensure a good start in a new country for refugee minors, because of their experiences of uprooting, potential trauma and flight. Easy access to good information and cultural guidance made the situation feel safer and more predictable. Integrational support, such as access to housing, money, work/education, language courses and interpreters, ensured basic needs were met and these helped to increase the possibility of independence. Providing support meant parents felt safe, and in turn managed to support their children. All participants stressed the importance of providing support very quickly after arrival as delays added stress and insecurity. They also meant that starting school or kindergarten soon after arrival to Norway would provide the children with the sense of returning to everyday life, normality, and the safety of routines. In addition – attending school meant days were filled with varied and meaningful activities, instead of the children being passive and waiting. In addition, they had opportunities to make new friends and play. Access to recreational activities outside school was also mentioned by many. Meaning and activity were identified as important factors by our participants, as passive waiting – although one might feel safe, they felt would not provide refugee minors with possibilities to grow and develop. Early contact with health services through routine assessments was considered as important to help uncover unmet health needs, potential trauma, provide access to treatment and referral if needed. Lastly, informants meant that a general reception climate which is positive to the arrival of refugees, was necessary for all these factors to be useful and experienced as positive by the children and their families. Frontline professionals’ opinion on the needs of refugee minors are visualized in Fig. 2.

A summary of these results was graded as accurate by participants in round 2. Several also commented on barriers to provide the support described above, such as lack of resources or qualified personnel, or disjointed services. In the focus group, these aspects were elaborated on by the participating PHNs. They explained that multiple practical and bureaucratic barriers related to a person’s settlement status meant that health personnel spent a lot of their available time doing cumbersome administrative work. As an example, the basic task of getting new glasses for a child, could involve booking interpreters, organizing transport, getting time off for parents on language courses, filling out forms to get the costs covered, all of which was complicated and time consuming. The participants suggested that simplifying these procedures could resolve modest health issues quickly, thus retaining resources to solve problems where their health competence is needed.

#### Competencies needed to meet the health needs of refugee minors

In round 1 participants suggested which competencies they meant were important to meet the needs of refugee minors, these were rated for importance in round 2 (see Table 4).

**Table 4:** Important competencies to meet the needs of refugee minors

Important competencies	Average score (1–10)
General communication and relationship skills	8.7
The national guidance on health services for newly arrived immigrants	8.7
Children’s development and health	8.6
Background as health professional	8.6
Vaccines and infection	8.5
Psychological health	8.3
Working with interpreters	7.8
Migration health	7.7
Trauma reactions	7.3
Intercultural competence	7.2
The health situation and challenges in countries of origin	7.0

The top four competences, with very similar rating, were ‘general communication skills’, a ‘health professional background’, ‘expertise in children’s health’ and ‘knowledge about the national guide’. In round 3, participants were surprised at the low rating of ‘migration health expertise’ and ‘trauma knowledge’ as they considered these essential competencies. They suggested that the low rating may be due to a lack of knowledge, believing that if one does not have knowledge on trauma reactions, one does not look for them or may misinterpret trauma reactions as behavior problems, stating that *“For me*,* trauma knowledge is essential”.* Others argued that general competencies, such as knowledge of children health and communication skills, were necessary as a base and by rating these generally high, specific competencies such as mental health or trauma knowledge automatically came far down on the list. They also meant that competencies were not enough, you also needed a genuine interest for the field of migration health, “*someone interested and curious as to what distinguishes migration health services from other health services”.*

Participants were also asked what was important to ensure good health services for refugee minors. Statements from round 1 were rated for importance in round 2 (see Table 5).

**Table 5:** Important factors to ensure good health services for refugee minors

Important factors	Average score (1–10)
Enough time	9.2
Good routines for cooperation and easy referral	9.0
Designated service/personnel for migration health	8.8
Close collaboration with doctors	8.6
Multidisciplinary teams coordinating the whole settlement process	8.3
Close collaboration with psychologist/psychosocial services	8.1
Standardized assessment forms	7.6
Materials adjusted for age	7.4
Materials in different languages	7.3
Standardized assessment forms available in different languages	6.8

Top rating was to have ‘enough time’, closely followed by ‘good routines for cooperation’ and ‘designated services or personnel for migration health’. The most prominent factors mentioned involved organization and cooperation, whilst translated information material and assessment tools came last. However, participants in round 3 highlighted the need for such tools; some had even developed their own, based on literature and their own experience. They also wanted better, more detailed and compulsory guidelines for children and youth with migration or refugee backgrounds, believing this would improve quality of services and reduce some of the differences between municipalities.

## Discussion

This study aimed to explore how health assessments among refugee minors, as described by the national guide on health services for newly arrived refugees, asylum seekers and reunited family members [16], were carried out in a Norwegian municipal setting. Results show that health assessments of children were predominantly conducted by PHNs, but the organisational structures surrounding assessments varied greatly according to the size of the municipalities and to how much resources were allocated locally. Reports from both the period before [24] and after [25] the recent influx of Ukrainian refugees show the same – large municipal variations in organisation, allocated resources and competence. According to our participants, most refugee minors are offered routine health assessments, and these are generally well attended. This is in line with a previous study showing that migrant children in most EU countries undergo regular health examinations [14]. Early health assessments are recommended as a universal intervention in the national guide [16], although, some respondents in our study only offered health assessments to families with extra needs or not at all. In addition, some municipalities differentiated assessments according to asylum status and did not include topics such as mental health, despite all children having the same right to health services [16]. There were also other differences in how the assessments were done - some went on home visits, others invited and assessed the whole family as a group, a third talked to the children alone, whilst others exclusively interacted with parents without involving the children. The experience of attending these assessments and what health needs that are uncovered, might therefore vary widely.

Internationally, health assessments for migrants focus largely on physical health, primarily infectious diseases [14], and this was also routinely addressed by our participants. However, studies on health in migrant children show that they also have mental health needs that are often undiscovered and unmet [11, 26]. In our study, general wellbeing and protective factors such as family and friend networks, were commonly addressed mental health topics. Our findings are in line with results of an Estonian study [27] in which minor Ukrainian refugees outlined the importance of family, peer support and engagement in leisure activities as facilitating their adaptation in the host country. Many also routinely assessed potentially traumatic experiences, sleep problems and mental distress, but it is unclear how these assessments were done. Many used the forms and questions provided in the national guide, but these are not intended for children. The lack of validated screening tools to assess trauma and mental health problems in refugee children is noted in research, especially for minor below the age of six years [28, 29]. Some of our participants missed having access to such tools and several had instead developed tools and questions of their own. Several also postponed mental health assessments of recent arrivals, arguing that potential mental distress required long-term relationships and follow-up and should therefore be done when the families had permanent settlement. However, the average stay in Norwegian reception centres is now more than one year and families with children and unaccompanied minors wait the longest for permanent resettlement in a municipality [24]. Other barriers to addressing mental health may be cultural stigma related to mental health problems or health personnel lacking trauma competence [13, 25]. Taken together, these factors could prevent mental health topics from being addressed appropriately, i.e. early enough, adequately and tailored to age and culture.

Although early health assessments are important, a multitude of factors long after arrival can greatly impact the health and wellbeing of refugee minors [7]. Everyday stressors such as language problems, economic concerns or discrimination may have greater long-term impact than trauma experiences, and delay recovery from trauma [30]. Hence, long-term support is important even when no serious health needs are uncovered in initial assessments. Some municipalities in this study provided long-term support from specialised refugee health services, but the majority transferred responsibilities to general CHS soon after the initial assessments, thereby including the children and youth in the national screening and vaccination programs. Depending on age, the next assessment offered might be years ahead. Despite potential health needs described in literature and the national guide, the current guide does not recommend any further assessments for refugee minors.

Unpredictable settlement conditions and waiting times are stressful for refugee minors [7] but also hindered transfer of health documentation for our participants. A lot of time was spent tracking test results, documents or referrals, often without success. Multiple tests and assessments therefore had to be repeated. Although recommended, very few provided a copy of the assessment to the parent, which they could have used to document testing or results. Missing health documentation is a recurring topic in literature, and perceived barriers should be explored in future studies. After the influx of Syrian refugees to Norway in 2015 the Norwegian Directorate for Health published a report describing several solutions to this problem – for example core journals or digital health cards for migrants [31], but none of these seemed to be implemented.

When describing the needs of refugee minors, our participants described early and good information and support as essential for safety. Our study showed that a considerable amount of the time reserved for assessments was spent providing general health information or solving practical problems for the families. A Norwegian report describes great potential in improving health information by targeted collaboration between sectors and organizations [32], which might leave health personnel with more time for the actual health assessments.

In this study we also explored the current needs of refugee minors in Norway, according to the participating professionals. They perceived the needs of refugee minors to be safety, provided through predictability and stability, but also opportunities for meaningful and positive activities such as learning and playing – closely resembling the descriptions in the national guide [16] and descriptions from refugee minors themselves [27]. The Adaptation and Development after Persecution and Trauma (ADAPT) model is a conceptual framework to underpin existing policies and practices, and the model suggest that five core psychosocial pillars are disrupted for refugees and restoring these are essential to reinstating psychosocial recovery [33]. The first pillar, Safety and Security, is fundamental to support natural recovery as well as providing services for acute reactions. The last pillar is Existential Meaning, which means re-establishing institutions and practices that confer meaning [33] – and these two pillars resemble our participants descriptions of refugee minors’ needs. The other pillars in the ADAPT model describe the importance of stable relationships, positive identities and feelings of acceptance, closely related to our participants descriptions of positive reception climates. Our participants suggested that to meet these needs, it was important to provide information, cultural guidance, support services, early enrolment in school and kindergarten upon arrival, engagement in leisure activities, and establishing early contact with health services. This reflects existing research indicating that refugee minor’s needs are primarily in the domains of social support, security, culture and education, thus conditions in resettlement countries play a crucial role in the overall resettlement process [34]. School environments have been recognized as safe and supportive, and positive attitudes from teachers and friends especially significant [35]. Additionally, participants actively engaging in their local communities through sports and recreational pursuits and the social support provided by refugee agencies and organizations, foster a sense of purpose and a strong connection to a community [36]. Health services must therefore not only provide early access but collaborate closely with other services to provide the necessary support. Good healthcare and low-threshold psychosocial services are crucial for the quality of life of refugees and can promote their ability to integrate later [37, 38].

Lastly, we wanted to examine the potential for improvement in health services for refugee minors. The participants rated sufficient time and resources and well-established routines for collaboration as the most important for providing a good service, reflecting the organisational challenges described above. Municipalities with designated services or personnel for migration health, found more often in the larger municipalities, seemed to have solved some of these problems. According to a systematic review from high-income countries, time, organisation and collaboration seem to be universal challenges for provision of primary healthcare for refugees [39]. The same review notes how health services are greatly affected by asylum and resettlement policies and practices, creating some of the unique challenges with discontinued care and unpredictability described by our participants.

Being a health professional and having good communications skills was seen as fundamental for providing a good service, coupled with knowledge on child development and the national guidance on healthcare for refugees. The participants also emphasized the necessity of guidelines, forms, and questions tailored for refugee minors, especially on mental health topics. Lastly, some suggested the development of distinct guidelines for refugee minors, pointing out that the child perspective was not addressed in the national guidelines for newly arrived refugees, and refugee minors were not adequately covered in the national guidelines for CHS [40]. Providing healthcare to children is different than for adults, and adjusted and better guidance might contribute to better services.

The theory of planned behaviour has been used to predict or explain the decisions and behaviour of health personnel by evaluating how three underlying factors influence (a) the intention to act, (b) the perceived behavioural control, and (c) the subjective norm and attitude [41]. Perceived behavioural control relates to whether individuals believe they have control in carrying out the action. Reflecting on our participants descriptions of practice, they often felt a lack of control. Larger social and political structures influenced refugee minors’ length of stay, rights and procedures, and a local lack of resources, routines and cooperation affected their scope of practice. The underlying factor of subjective norm relates to the perceived social pressure from important others and could, among our participants, be the influence of norm documents such as the national guidance. Most of our informants followed the guidance, but with various interpretations in practice. Lastly, attitude relates to the belief an individual holds towards the action. This aspect was little mentioned by our participants but might be reflected in descriptions of “a positive reception climate” and also the sentiment that knowledge is not enough to provide a good service for refugee minors, they felt that health professionals need to have a genuine interest for the field of migration health.

A European study revealed that the lack of evaluation of health assessment programs directed towards migrants, despite substantial investments, raises concerns regarding their effectiveness in addressing the healthcare needs of migrants [14]. As a first step toward evaluating and improving health services, this study has attempted to describe what health professionals perceive to be the needs of refugee minors, and how health assessments are carried out.

### Strength and limitations

To our knowledge, this is the first study which applies a Delphi approach, exploring health assessment practice among refugee minors in Norway. Knowing little about who the “frontline professionals” were, how responsibilities were shared and how assessments were done and documented, the Delphi approach was a pragmatic choice which allowed for potential diversity, whilst reaching a consensus on main content, needs and challenges. Other potential methods such as ethnography, accessing health records or purely quantitative measures were considered and could have provided more detailed answers, but may have been unreliable as it was not clear who would be the right person to approach, no registers available to draw a representative sample from, nor did we know what the appropriate questions were. The Delphi method allowed for this diversity and the possibility of describing a variety of practice, whilst reaching a consensus on needs, challenges, and tools. However, the result of this study may not necessarily reflect the actual practice everywhere, it merely helps to identify areas that the participants consider important in relation to health assessment of minor refugees. With this modified Delphi approach, we did not reach all relevant stakeholder in the municipalities. The presented data only includes those responding, which affects generalisation. Due to data protection, we could not identify if the respondents in round 2 were exactly the same as in round 1.

### Conclusions and implications

This study explored the current practice of early health assessments of refugee minors, frontline professionals’ opinions on their needs, and potentials for improvement. The study showed that the majority of municipalities invite refugee minors and their families to early health assessments, but that there is a great variety in organization and collaboration. A wide range of health topics are addressed in the assessments and families are offered information and practical help. However, there is room for improvement in several areas: A substantial group is not offered assessments, some topics are not routinely addressed or postponed, missing documentation and practical barriers impede quality and take time away from assessments. Most variation seems to be in mental health assessments. The participants in this study perceived the main needs of refugee minors to be safety and stability, combined with meaningful activities, underscoring the importance of coordinated effort from several services. Professionals themselves felt that more time, better organisation and co-operation are the most crucial aspects to improve the quality of services. In addition, participants wanted better competency in migration health and age-adjusted guidelines to be able to meet the needs of refugee minors. Based on these findings, future studies can explore current practice in more detail, but more importantly – the experience of refugee minors of attending these health assessments.

## Data Availability

Data (results from the online questionnaire and transcripts of the focus group) from this Delphi study are available on request to the authors.

## Footnotes

1 PHN (Foot note) Public Health Nurses in Norway have additional training in public health, health promotion, preventive care and development in children and youth (0–20 years) (minimum 60 ECT) and are the primary health personnel in municipal Child Health Services (CHS).

## Abbreviations

CHS: Child Health Services
PHN: Public Health Nurse
